# LAMP3 transfer via extracellular particles induces apoptosis in Sjögren’s disease

**DOI:** 10.1038/s41598-023-28857-w

**Published:** 2023-02-14

**Authors:** Tsutomu Tanaka, Hiroyuki Nakamura, Duy T. Tran, Blake M. Warner, Yan Wang, Tatsuya Atsumi, Masayuki Noguchi, John A. Chiorini

**Affiliations:** 1 Adeno-Associated Virus Biology Section, National Institute of Dental and Craniofacial Research, National Institutes of Health, 10 Center Drive, Bethesda, MD 20892 USA; 2 NIDCR Imaging Core, National Institute of Dental and Craniofacial Research, National Institutes of Health, Bethesda, MD USA; 3 Salivary Disorders Unit, National Institute of Dental and Craniofacial Research, National Institutes of Health, Bethesda, MD USA; 4 Mass Spectrometry Facility, National Institute of Dental and Craniofacial Research, National Institutes of Health, Bethesda, MD USA; 5 Department of Rheumatology, Endocrinology and Nephrology, Faculty of Medicine and Graduate School of Medicine, Hokkaido University, Sapporo, Japan; 6 Division of Cancer Biology, Institute for Genetic Medicine, Hokkaido University, Sapporo, Japan

**Keywords:** Cell biology, SjÃ¶gren's disease

## Abstract

Sjögren’s disease (SjD) is an autoimmune disease that affects exocrine tissues and is characterized by increased apoptosis in salivary and lacrimal glands. Although the pathogenic mechanism triggering SjD is not well understood, overexpression of lysosome-associated membrane protein 3 (LAMP3) is associated with the disease in a subset of SjD patients and the development of SjD-like phenotype in mice. In this study, histological analysis of minor salivary glands of SjD patients suggested that LAMP3-containing material is being ejected from cells. Follow-on in vitro experiments with cells exposed to extracellular particles (EPs) derived from LAMP3-overexpressing cells showed increased apoptosis. Proteomics identified LAMP3 as a major component of EPs derived from LAMP3-overexpressing cells. Live-cell imaging visualized release and uptake of LAMP3-containing EPs from LAMP3-overexpressing cells to naïve cells. Furthermore, experiments with recombinant LAMP3 protein alone or complexed with Xfect protein transfection reagent demonstrated that internalization of LAMP3 was required for apoptosis in a caspase-dependent pathway. Taken together, we identified a new role for extracellular LAMP3 in cell-to-cell communication via EPs, which provides further support for targeting LAMP3 as a therapeutic approach in SjD.

## Introduction

Sjögren’s disease (SjD) is a chronic autoimmune inflammatory disease that involves damage to salivary and lacrimal glands^1^. Although the exact etiology is still unclear, in salivary gland epithelial cells of SjD patients, as well as in mouse models of the disease, apoptosis has been documented^2,3^.

Previously, we reported lysosome-associated membrane protein 3 (LAMP3/CD208/DC-LAMP) can induce apoptosis in salivary gland epithelial cells^4^. Unlike other members of the LAMP family, which are ubiquitously expressed, LAMP3 expression is restricted to dendritic cells and type II pneumocytes^5,6^. LAMP3 is a direct transcriptional target of activating transcription factor 4 as part of the unfolded protein response pathway and is induced by endoplasmic reticulum stress following irradiation or infection^7–9^. Other research has shown elevated LAMP3 expression in response to interferon stimulation^10,11^. In the context of SjD, overexpression of LAMP3 in salivary glands of patients and mice is associated with autoantibody formation^4,12^. In additional experiments, LAMP3 expression was shown to be associated with increased rates of apoptosis via lysosomal membrane permeabilization (LMP), autoantigen release, decreased salivary gland activity, and immune cell activation^4,12–14^. LAMP3 expression also induces decreased expression of cell surface molecules, such as Na–K–Cl cotransporter-1 and aquaporin 5, via induced protein degradation, which results in salivary gland hypofunction in mice^12^. Furthermore, damage-associated molecular patterns released from LAMP3-overexpressing salivary gland epithelial cells stimulate toll-like receptors and activate monocytes/macrophages^12,14^.

Extracellular particles (EPs) exist throughout the body and can be separated from saliva, blood, amniotic fluid, milk, and urine^15–17^. They may contain DNA, mRNA, microRNA (miRNA), and proteins, which are likely to reflect the pathophysiological status of the cell of origin^17,18^. The release of cellular contents into EPs is an important form of cell-to-cell communication^19,20^.

For example, EPs released from infiltrating B cells in minor salivary glands (MSGs) of SjD patients are reported to contain Epstein Barr virus–specific miRNA (ebv-miR-BART13-3p). This miRNA is taken up by epithelial cells in MSGs, resulting in salivary gland exocrinopathy by inhibition of proteins that are important for salivary gland function^21^. Recent work has also suggested that miRNA in EPs released from activated T cells can impair the secretory function of salivary gland epithelial cells^22^.

In the current study, the role of LAMP3-associated EPs in cell-to-cell communication was investigated. We observed an increased rate of apoptosis in LAMP3-naïve wild type cells treated with the culture supernatant of LAMP3-overexpressing cells. Analysis of this supernatant showed that the increased apoptosis rate was associated with the EPs and that this cell death was endogenous caspase-dependent. Proteomics and Western blotting analysis following fractionation of the EPs identified LAMP3 as a major component of the EPs. Treatment with EPs from stably LAMP3-overexpressing cells or transfection with recombinant LAMP3 protein could also induce apoptosis in naïve cells. These findings suggest a new mechanism of cell-to-cell communication and damage to the salivary glands in SjD.

## Results

### Culture medium of LAMP3-overexpressing cells induces apoptosis in naïve cells

**Figure 1:**
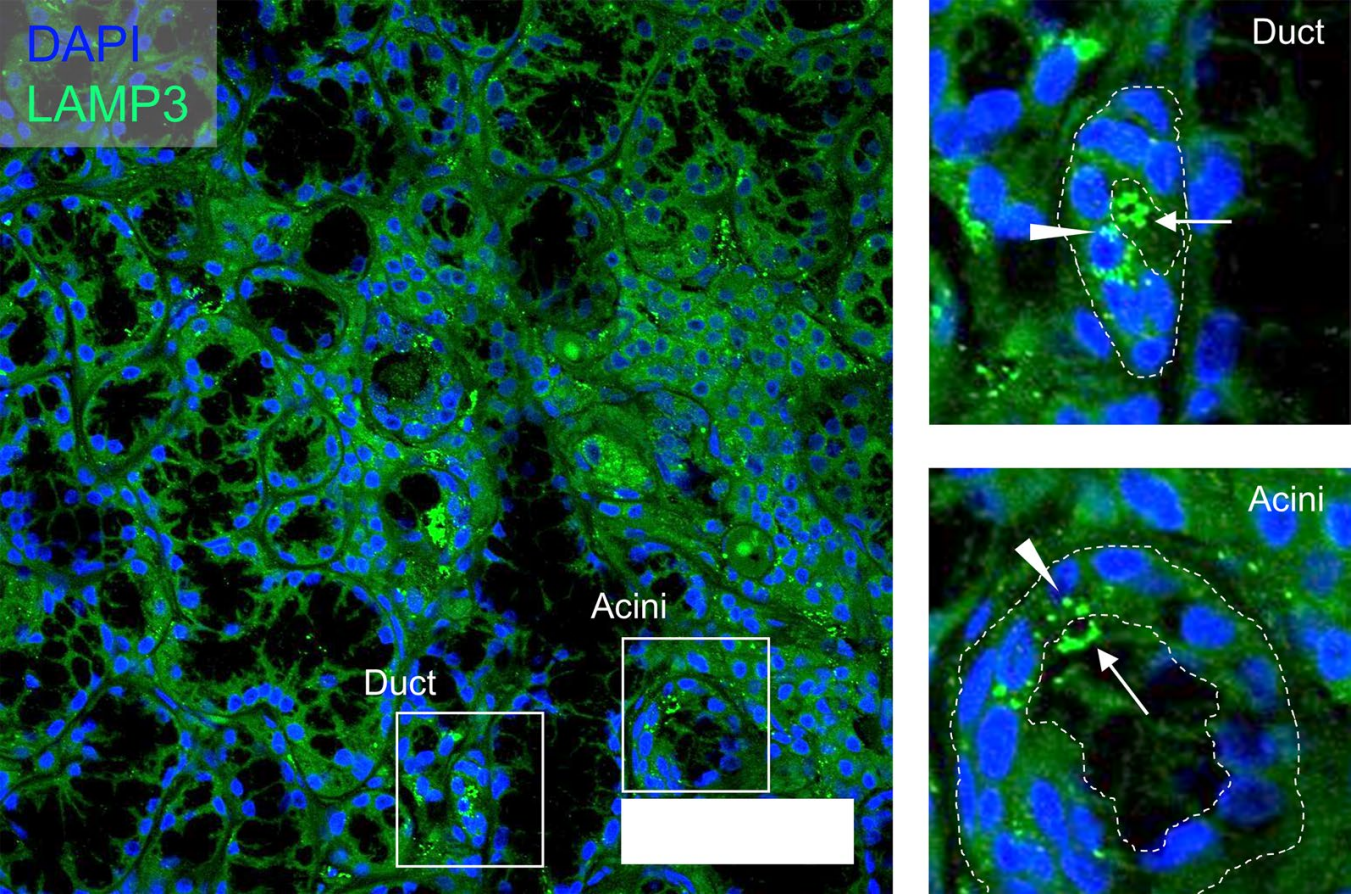
LAMP3-positive material is released in minor salivary glands from SjD patients. A representative immunofluorescent image of minor salivary glands from SjD patients (*n* = 11) stained with anti-LAMP3 antibody (scale bar: 50 μm). The image shows accumulation of LAMP3 in the lumen of the gland (the arrows) and in the epithelial cells (the arrowheads).

LAMP3 expression is elevated in MSGs of SjD patients compared with controls^4^. Examination of immunofluorescent confocal images of LAMP3 in MSGs from SjD patients showed that much of LAMP3 was accumulated in ductal and acinar cells. LAMP3-positive extracellular material could also be detected in the lumen of the glands and alongside the extracellular matrix on the basolateral surface of the cells, suggesting release of the LAMP3-positive material from the cells for cell-to-cell communication (Fig. 1).

**Figure 2:**
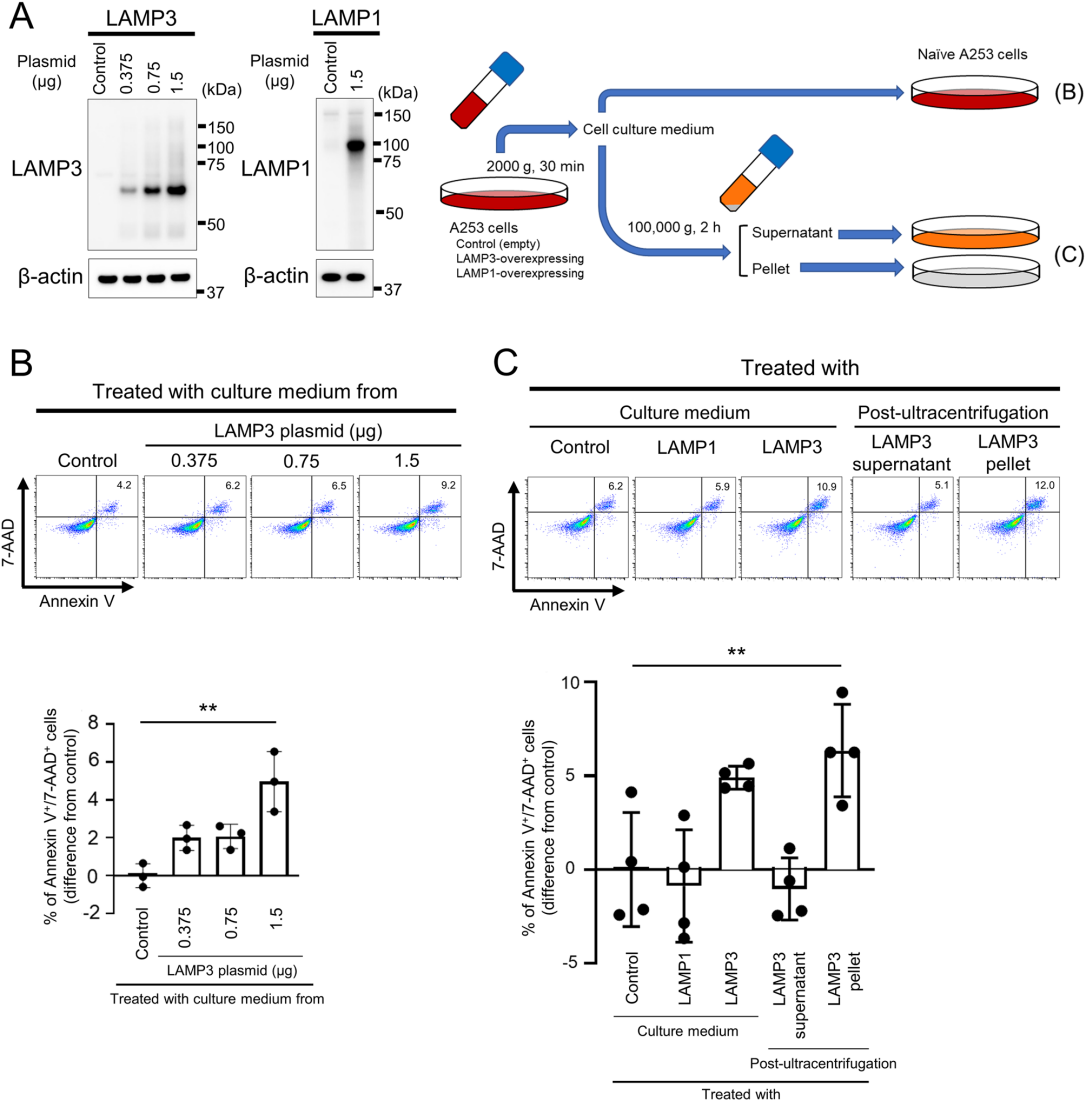
Culture medium of LAMP3-overexpressing A253 cells induce apoptosis in naïve A253 cells. A253 cells were transfected with control empty plasmid, *LAMP3*-encoding plasmid or *LAMP1*-encoding plasmid. (**A**) Representative Western blots of transfected A253 cells (uncropped images are provided in Supplementary Fig. 3) and schematic methods of the following in vitro assays. (**B**) Naïve A253 cells were treated with culture medium of control or LAMP3-overexpressing A253 cells for 48 h (*n* = 3). (**C**) Naïve A253 cells were treated with culture medium of control, LAMP1-overexpressing, or LAMP3-overexpressing A253 cells or with post-ultracentrifugation supernatant or post-ultracentrifugation pellet from LAMP3-overexpressing A253 cells for 48 h (*n* = 4). Apoptotic cells in naïve cell culture were determined by flow cytometry using APC Annexin V/7-AAD. Graphs show difference in mean (± SD) number of Annexin V^+^/7-AAD^+^ cells in cell culture compared with control. **p < 0.01 (one-way ANOVA).

LAMP3 overexpression induces apoptosis^4^. Since cells undergoing apoptosis release different proteins from their steady state^23^, we hypothesized that LAMP3-associated protein release and the released factors can induce apoptosis via altered cell-to-cell communication. To investigate the effect of released proteins from LAMP3-overexpressing cells on LAMP3-naïve cells, A253 cells were either transfected with *LAMP3*-encoding plasmid or as control *LAMP1*-encoding plasmid as well as empty plasmid and were used to treat LAMP3-naïve A253 cells (Fig. 2A). Analysis of the number of apoptotic cells in naïve A253 cell culture treated with the culture medium of LAMP3-overexpressing A253 cells showed a statistically significant 5.8% increase in apoptosis in naïve cells compared with the cells treated with the culture medium of control A253 cells (Fig. 2B). These results showed that LAMP3-overexpressing cells in vitro release factor(s) into the culture medium that is capable of inducing apoptosis in naïve cells.

Cell culture medium contains many soluble factors such as lysosomal enzymes, cytokines, chemokines, soluble receptors, miRNA, DNA, and EPs, which can all play a role in apoptosis. To examine which factors in the culture medium are associated with apoptosis in naïve cells, cell culture medium of LAMP3-overexpressing A253 cells was separated by ultracentrifugation into a pellet fraction and a supernatant fraction. Naïve A253 cells treated with the supernatant fraction excluding the pellet showed no increase in apoptosis, although naïve cells treated with the unfractionated culture medium of LAMP3-overexpressing cells showed the increase in apoptosis compared with cells treated with that of control and LAMP1-overexpressing cells (Fig. 2C). In contrast, treatment of naïve cells with the resuspended pellet fraction showed a significant increase in apoptosis comparable with treatment with unfractionated cell culture medium (Fig. 2C), suggesting the pellet fraction contained factor(s) responsible for inducing apoptosis in treated naïve cells.

### A fraction of extracellular particles derived from LAMP3-overexpressing cells induces apoptosis

**Figure 3:**
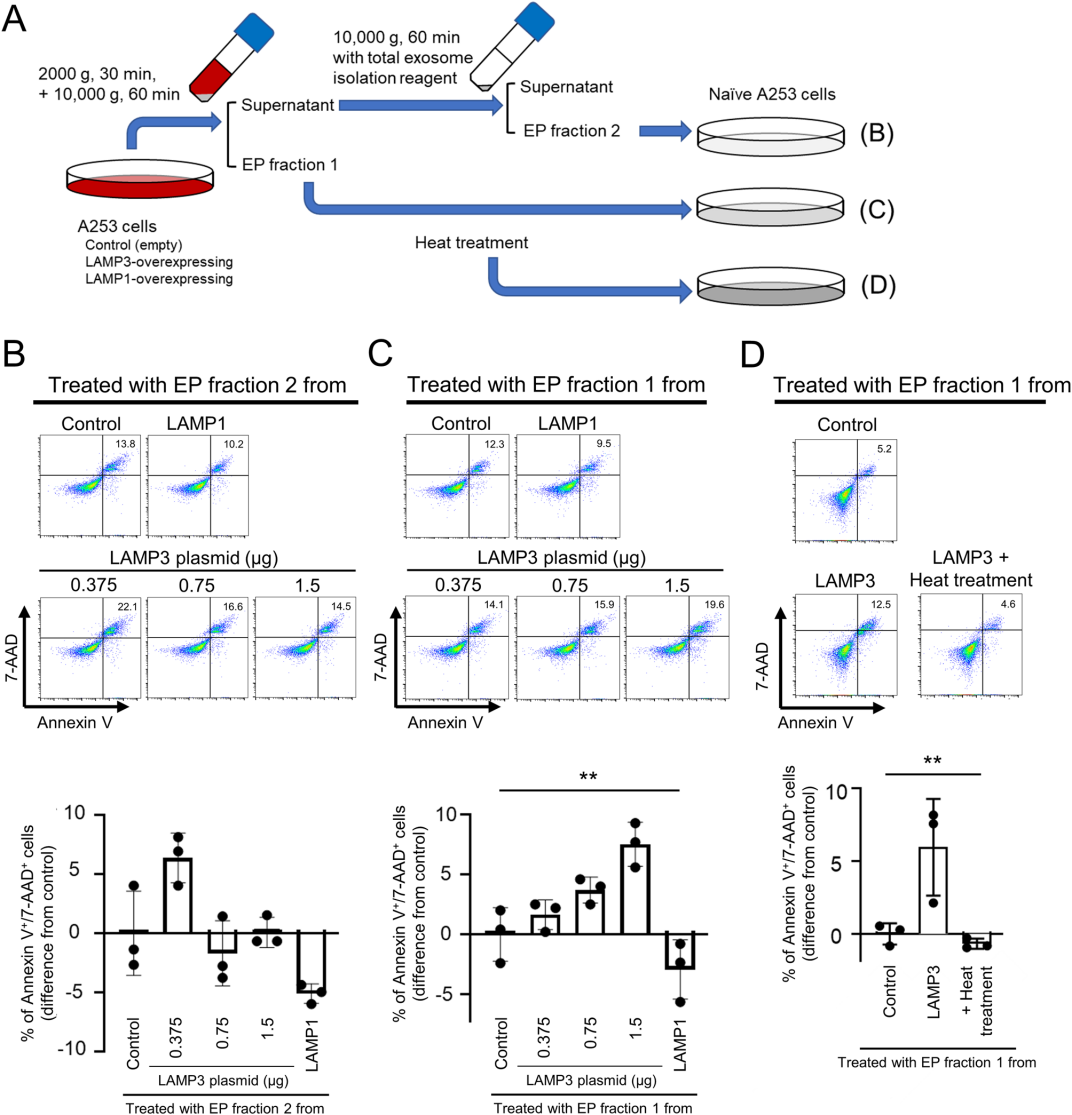
A fraction of extracellular particles from LAMP3-overexpressing A253 cells induces apoptosis in naïve A253 cells. (**A**) Schematic methods. Two fractions of extracellular particles (EPs) were separated from culture medium of control, LAMP3-overexpressing, or LAMP1-overexpressing A253 cells using centrifugation in combination with or without total exosome isolation reagent. Naïve A253 cells were treated with (**B**) EP fraction 2, (**C**) EP fraction 1 or (**D**) heat-denatured (heat-treated) EP fraction 1. Apoptotic cells in naïve cell culture were determined by flow cytometry using APC Annexin V/7-AAD 72 h after incubation. Graphs show difference in mean (± SD) number of Annexin V^+^/7-AAD^+^ cells in cell culture compared with control (*n* = 3 for all the experiments). **p < 0.01 (one-way ANOVA).

The pellet fraction contains EPs. Two fractions of EPs were separated from culture medium using differential centrifugation and specific isolation reagents (Fig. 3A)^24–26^. Although no significant increase in apoptosis was found in naïve A253 cells treated with EP fraction 2 (EP-f2) collected from the culture medium of LAMP3-overexpressing A253 cells compared those of control cells (Fig. 3B), treatment of naïve cells with corresponding EP fraction 1 (EP-f1) induced a significant dose-dependent increase in apoptosis in naïve A253 cells compared with those from control or LAMP1-overexpressing A253 cells (Fig. 3C). To characterize the EP-f1 component(s) responsible for this effect, separated EP-f1 were denatured by heating to 100 ℃ and tested for their potency in inducing apoptosis^27^. Heat treatment completely inhibited the apoptosis in naïve A253 cells that had been induced by the EP-f1 derived from LAMP3-overexpressing A253 cells (Fig. 3D).

LAMP3 overexpression induces apoptosis^4,13^. To determine whether the increased apoptosis in naïve cells is associated with apoptosis in EP producer cells, naïve A253 cells were treated with EPs from culture medium of A253 cells treated with staurosporine (STS), which is a general apoptosis inducer and is reported not to induce *LAMP3* mRNA expression^28^. As expected, STS treatment significantly increased apoptosis in A253 cells; however, treatment with EPs from STS-treated cells showed no significant increase in apoptosis (Supplementary Fig. 1). These results suggested that apoptosis induced by LAMP3-associated EPs is not due to apoptosis in EP producer cells, but is likely related to the component(s) in EPs specifically derived from LAMP3-overexpressing cells.

### Extracellular particles from LAMP3-overexpressing cells contain LAMP3

**Figure 4:**
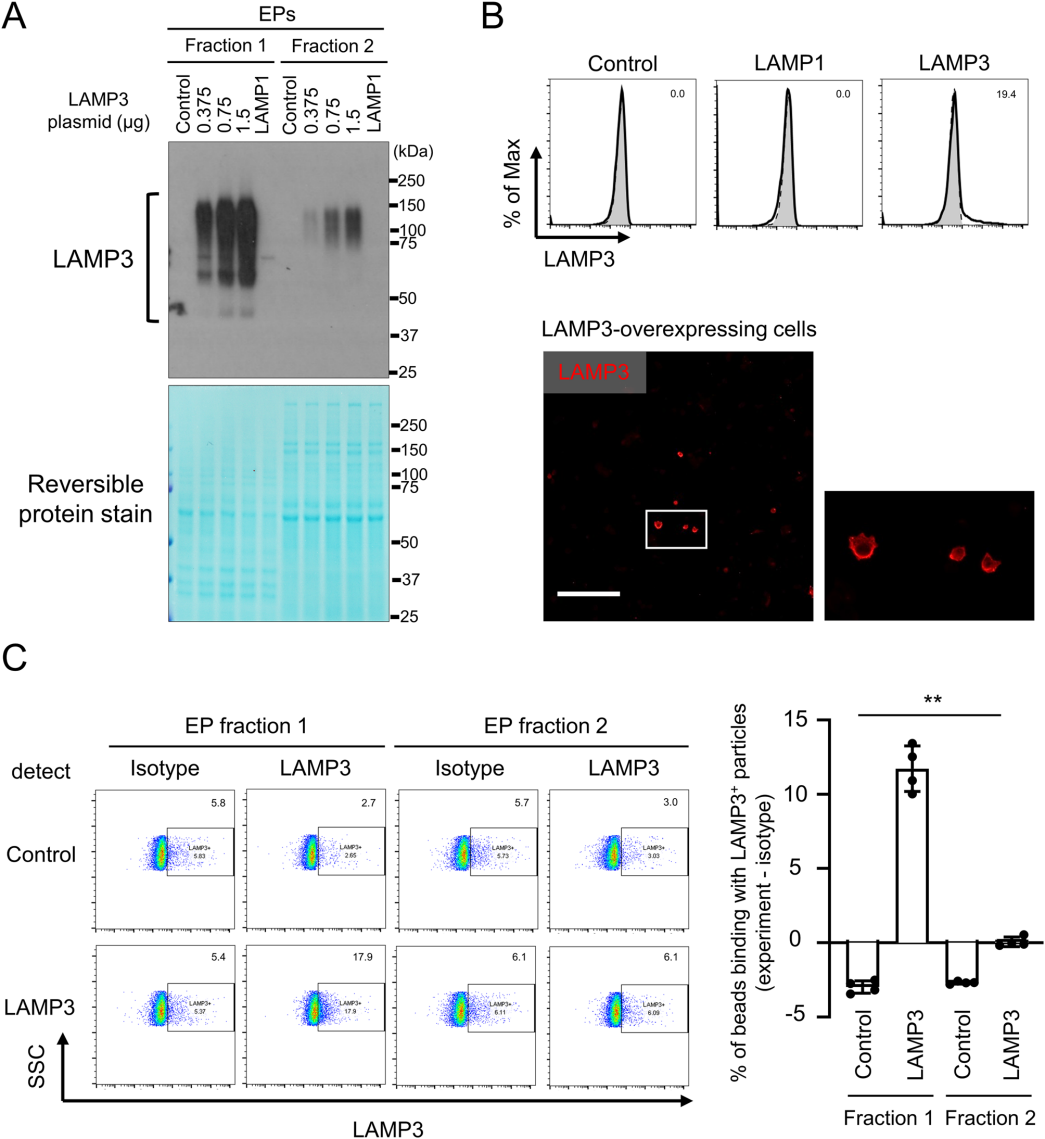
Extracellular particles from LAMP3-overexpressing cells contain LAMP3. (**A**) Western blot analysis of EPs separated from the culture medium of control, LAMP3-overexpressing, and LAMP1-overexpressing A253 cells using centrifugation in combination with or without total exosome isolation reagent. Equal volume of protein loaded in each lane. Lower panel shows same membrane as the one used in Western blot analysis but stained with Reversible Protein Stain Kit. Uncropped images are provided in Supplementary Fig. 3. (**B**) Cell surface LAMP3 expression was analyzed by flow cytometry (bold line: LAMP3 staining, filled area with dashed line: isotype control) and under an immunofluorescent microscopy (scale bar: 100 μm) in control, LAMP1-overexpressing, and LAMP3-overexpressing A253 cells 48 h post-transfection. (**C**) EPs derived from control and LAMP3-overexpressing A253 cells were immunoprecipitated using anti-LAMP3 monoclonal antibody-conjugated beads. Presence of LAMP3 protein on EP membrane surface was determined by analyzing beads stained with anti-LAMP3 polyclonal antibody using flow cytometry. Graph showing difference in mean (± SD) percentage of LAMP3-positive beads compared with beads bound to isotype control (*n* = 4). **p < 0.01 (one-way ANOVA).

To better understand the mechanism of action associated with the apoptosis which we observed in naïve cells induced by EP-f1 from LAMP3-overexpressing cells, RNA and protein content of EPs from LAMP3-overexpressing A253 cells was analyzed by RNA sequencing and proteomics. With RNA sequencing analysis, we were able to detect a wide range of abundance of miRNAs and mRNAs in EPs from LAMP3-overexpressing cells. Upon further analysis, the most differentially expressed genes (> twofold increase and *p* < 0.05) had read per million values less than 10, and could not be confirmed by qRT-PCR (data not shown). Proteomic analysis showed abundance of EP marker proteins, such as CD9, CD63, CD81, TSG101, ALIX, and calnexin, in EP-f1 (Supplementary Table 1). Additional analysis of the proteomic data showed that LAMP3 was abundantly contained in EP-f1 from LAMP3-overexpressing cells compared with EP-f1 from LAMP1-overexpressing cells and EP-f2 from LAMP1-overexpressing or LAMP3-overexppressing cells (Supplementary Table 2), which was confirmed by Western blotting (Fig. 4A). These results suggested that LAMP3 protein in the EP-f1 was related to increased apoptosis in treated naïve cells.

Because EPs are formed by plasma membrane, some plasma membrane proteins, such as major histocompatibility complex class I, remain on the surface of EPs^29,30^. Presence of cell surface LAMP3 expression was shown by flow cytometric and microscopic analysis in LAMP3-overexpressing A253 cells (Fig. 4B). Flow cytometric analysis indicated that LAMP3 expression was found on the membrane surface of EP-f1 from LAMP3-overexpressing A253 cells (Fig. 4C).

### Extracellular particles from LAMP3-overexpressing cells transfer LAMP3 to naïve cells.

**Figure 5:**
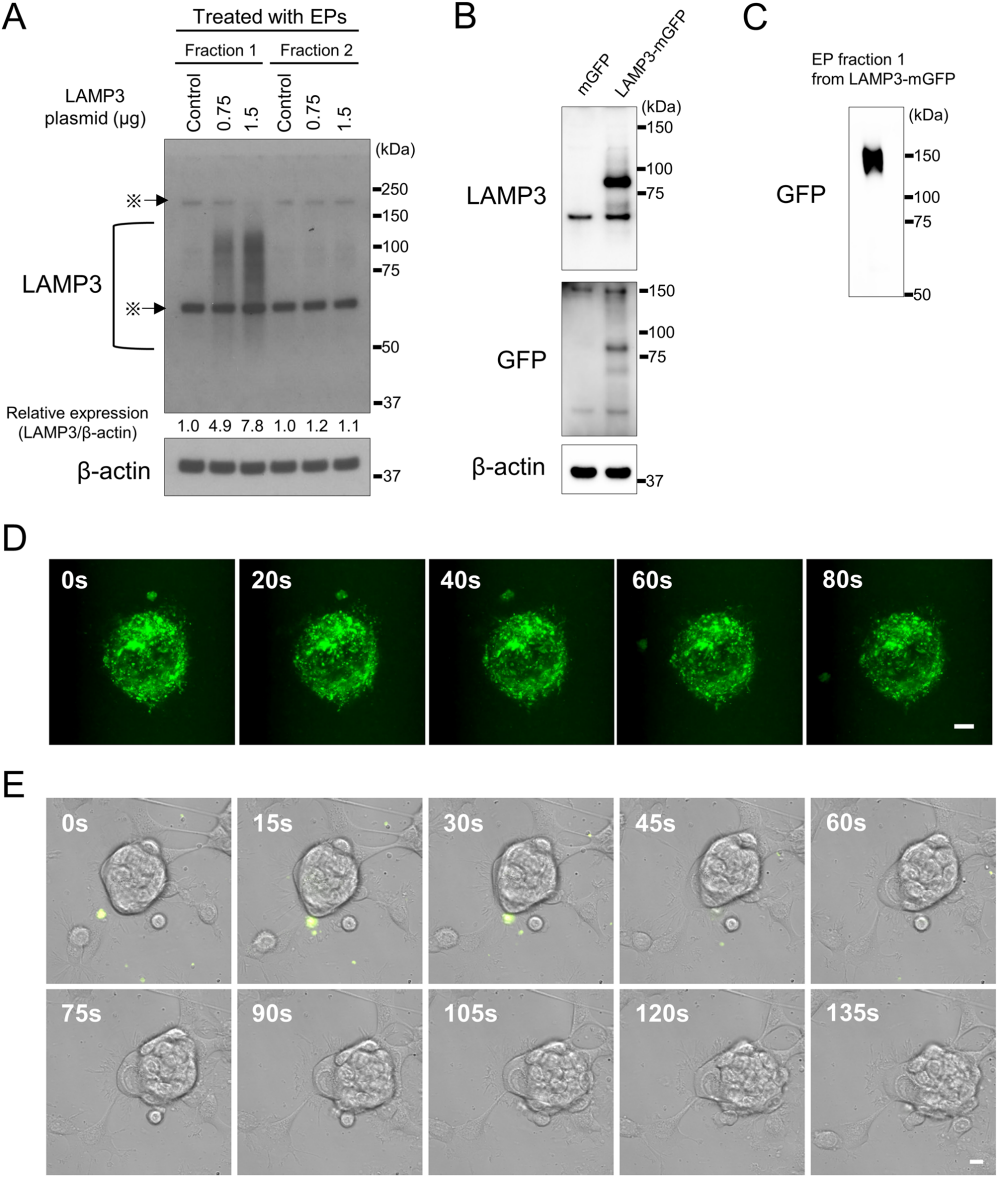
Extracellular particles from LAMP3-overexpressing transfer LAMP3 to naïve cells. (**A**) Western blot analysis of naïve A253 cells treated with EPs from control or LAMP3-overexpressing A253 cells. “※” indicates non-specific bands. (**B**) Western blot analysis of LAMP3-GFP expression in A253 cells transfected with empty plasmid (control) or pLenti-LAMP3-mGFP plasmid (A253-LAMP3-mGFP cells). (**C**) Western blot analysis of EPs derived from A253-LAMP3-mGFP cells. (**D**) A253-LAMP3-mGFP cells were imaged in real time to visualize EP release. A particle containing LAMP3-mGFP is seen being released from the cell into the extracellular environment (scale bar: 5 μm). (**E**) Naïve A253 cells were treated with LAMP3-mGFP–containing particle and imaged in real time to visualize particle uptake. Uptake of a LAMP3-mGFP–containing particle by a naïve cell and subsequent membrane blebbing are shown (scale bar: 10 μm). Uncropped images of Western blots are provided in Supplementary Fig. 3.

To study whether LAMP3 protein was transferred into naïve cells via LAMP3-containing EPs, LAMP3 expression levels were compared in naïve A253 cells treated with EPs derived from control or LAMP3-overexpressing A253 cells. Western blot analysis of naïve A253 cells treated with EP-f1 from LAMP3-overexpressing A253 cells showed a dose-dependent increase in LAMP3 expression (Fig. 5A).

For further confirmation, LAMP3 protein transfer was monitored by live-cell imaging using A253 cells stably overexpressing LAMP3-mGFP (A253-LAMP3-mGFP cells) (Fig. 5B). A253-LAMP3-mGFP cells released LAMP3-mGFP protein via an EP into the extracellular environment (Fig. 5C,D and Supplementary Video 1). Treatment of naïve A253 cells with LAMP3-mGFP–containing particles showed cellular uptake of a particle into cytosol followed by blebbing, which suggested the start of apoptosis (Fig. 5E and Supplementary Video 2). These results supported our finding that LAMP3 protein in EPs could be transferred from LAMP3-overexpressing cells to naïve cells and thereafter initiate apoptosis.

### LAMP3 protein transfer induces apoptosis via a caspase-dependent pathway

The above results suggested that transferred LAMP3 protein was required for inducing apoptosis in naïve cells via LAMP3-associated EPs. Because EPs from LAMP3-overexpressing cells contain *LAMP3* mRNA as well as LAMP3 protein, the assay was repeated using EPs derived from A253 cells stably overexpressing LAMP3 (A253-LAMP3 cells) (Supplementary Fig. 2A). Naïve A253 cells showed similar patterns of apoptosis following treatment with EPs derived from A253-LAMP3 cells (Supplementary Fig. 2B).

**Figure 6:**
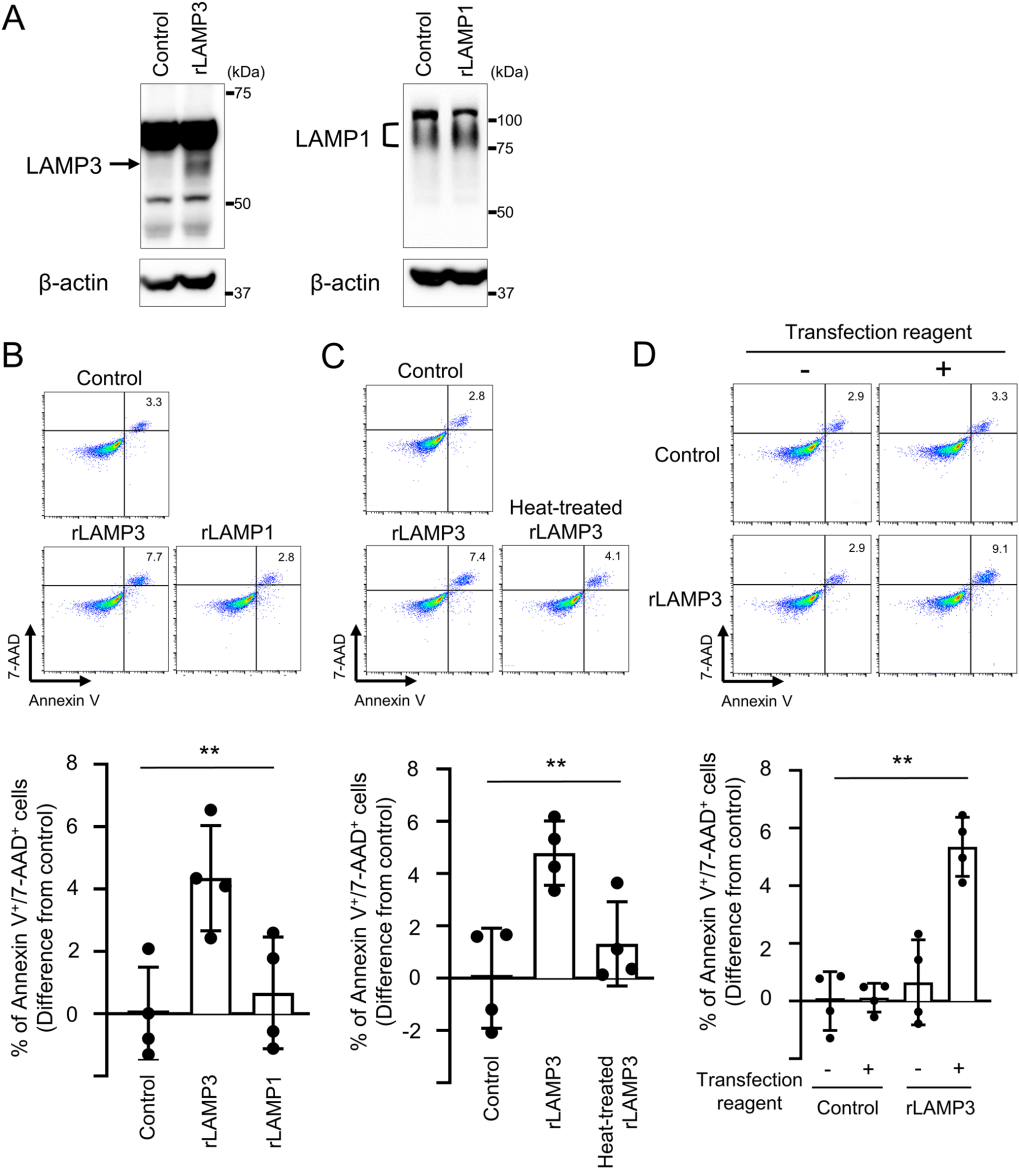
Transfection of recombinant LAMP3 protein induces apoptosis in A253 cells. (**A**) Representative Western blots of A253 cells transfected with recombinant LAMP3 (rLAMP3), recombinant LAMP1 (rLAMP1), or control FLAG peptide (uncropped images are provided in Supplementary Fig. 3). (**B**) Apoptotic cells were determined in A253 cell culture by flow cytometry using APC Annexin V/7-AAD 14 h after transfection. Graph showing difference in mean (± SD) number of Annexin V^+^/7-AAD^+^ cells in A253 cell culture transfected with rLAMP3 or rLAMP1 compared with control. (**C**) A253 cells were transfected with rLAMP3, heat-treated rLAMP3, or FLAG peptide. Apoptotic cells were determined in A253 cell culture by flow cytometry using APC Annexin V/7-AAD 14 h after transfection. Graph showing difference in mean (± SD) number of Annexin V^+^/7-AAD^+^ cells in A253 cell culture transfected with rLAMP3 or heat-treated rLAMP3 compared with control. (**D**) A253 cells were treated with control FLAG peptide or rLAMP3 alone (without transfection reagent) or transfected with control FLAG peptide or rLAMP3 with transection reagent. After 14 h, number of apoptotic cells was determined by flow cytometry using APC Annexin V/7-AAD. Graph showing difference in mean (± SD) number of Annexin V + /7-AAD + cells in cell culture treated with rLAMP3 alone or transfected with FLAG peptide or rLAMP3 compared with FLAG peptide alone. **p < 0.01 (one-way ANOVA, *n* = 4 for all the experiments).

In addition, naïve A253 cells were transfected with recombinant LAMP3 (rLAMP3), recombinant LAMP1 (rLAMP1) or control FLAG peptide and analyzed for apoptotic events using flow cytometry (Fig. 6A). Compared with FLAG peptide-transfected and rLAMP1-transfected A253 cells, rLAMP3-transfected A253 cells showed a significant increase in apoptosis (Fig. 6B). No significant increase in apoptosis was found in A253 cells transfected with heat-treated rLAMP3 (Fig. 6C). Similarly, no significant increase in apoptosis was observed by adding rLAMP3 without transfection reagent (Fig. 6D). Taken together, these results suggested that LAMP3 protein transfer could induce apoptosis.

**Figure 7:**
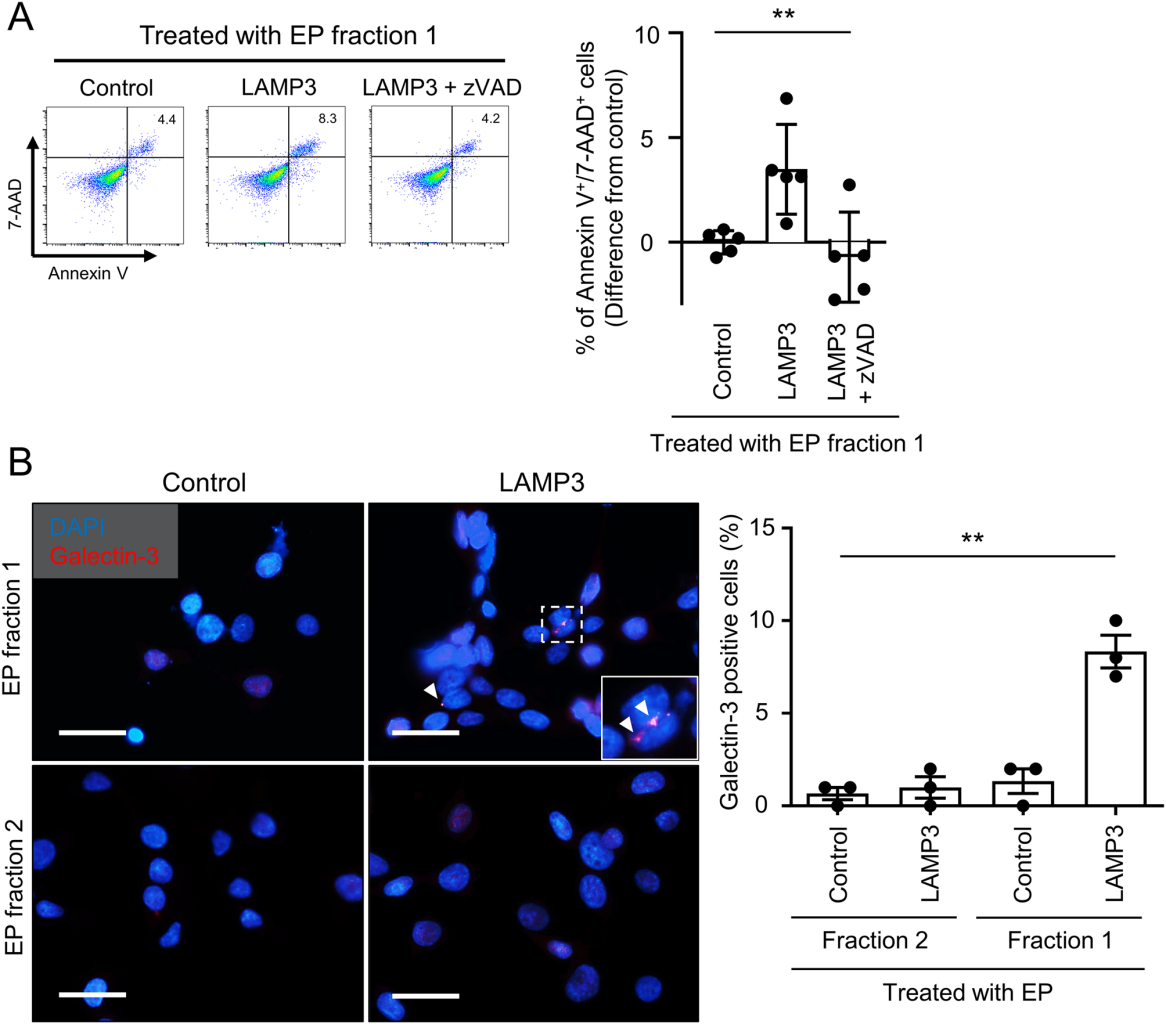
LAMP3 transfer via extracellular particles induces caspase-dependent apoptosis. (**A**) Naïve A253 cells were treated with EPs from control and LAMP3-overexpressing A253 cells in combination with or without zVAD-fmk (zVAD), a pan-caspase inhibitor. Apoptotic cells were determined by flow cytometry using APC Annexin V/7-AAD 72 h after incubation. Graph showing difference in mean (± SD) number of Annexin V^+^/7-AAD^+^ cells in naïve A253 cell culture compared with control (*n* = 5). (**B**) Naïve A253 cells were treated with EPs from control and LAMP3-overexpressing A253 cells for 6 h. Galectin-3 puncta formation in treated A253 cell culture was visualized by immunofluorescence (scale bar: 50 μm). Graph showing percentage of galectin-3 puncta–positive cells per 100 treated A253 cells (*n* = 3). **p < 0.01 (one-way ANOVA).

LAMP3-overexpressing cells established by transfection with *LAMP3*-encoding plasmid induces apoptosis via a caspase-dependent pathway following LMP^13^. To test whether LAMP3 protein transfer through EPs induced apoptosis via the caspase-dependent pathway, naïve A253 cells were treated with EPs in combination with zVAD-fmk, a pan-caspase inhibitor. Treatment with zVAD-fmk significantly inhibited apoptosis induced by LAMP3-containing EPs (Fig. 7A).

Lysosomal membrane integrity of the cells treated with EPs from control or LAMP3-overexpressing A253 cells was evaluated using galectin-3 staining. Galectin-3 puncta formation indicates a loss of lysosomal membrane integrity as a marker of LMP, which may imply release of lysosomal digestive enzymes into the cell cytoplasm^31^. The percentage of galectin-3 puncta-positive cells in the A253 cells treated with EP-f1 from LAMP3-overexpressing A253 cells was significantly increased compared with EPs from control cells (Fig. 7B). These results demonstrated that LAMP3-containing EPs induced caspase-dependent apoptotic cell death following LMP, similar to the result obtained in *LAMP3*-encoding plasmid-transfected cells^13^.

**Figure 8:**
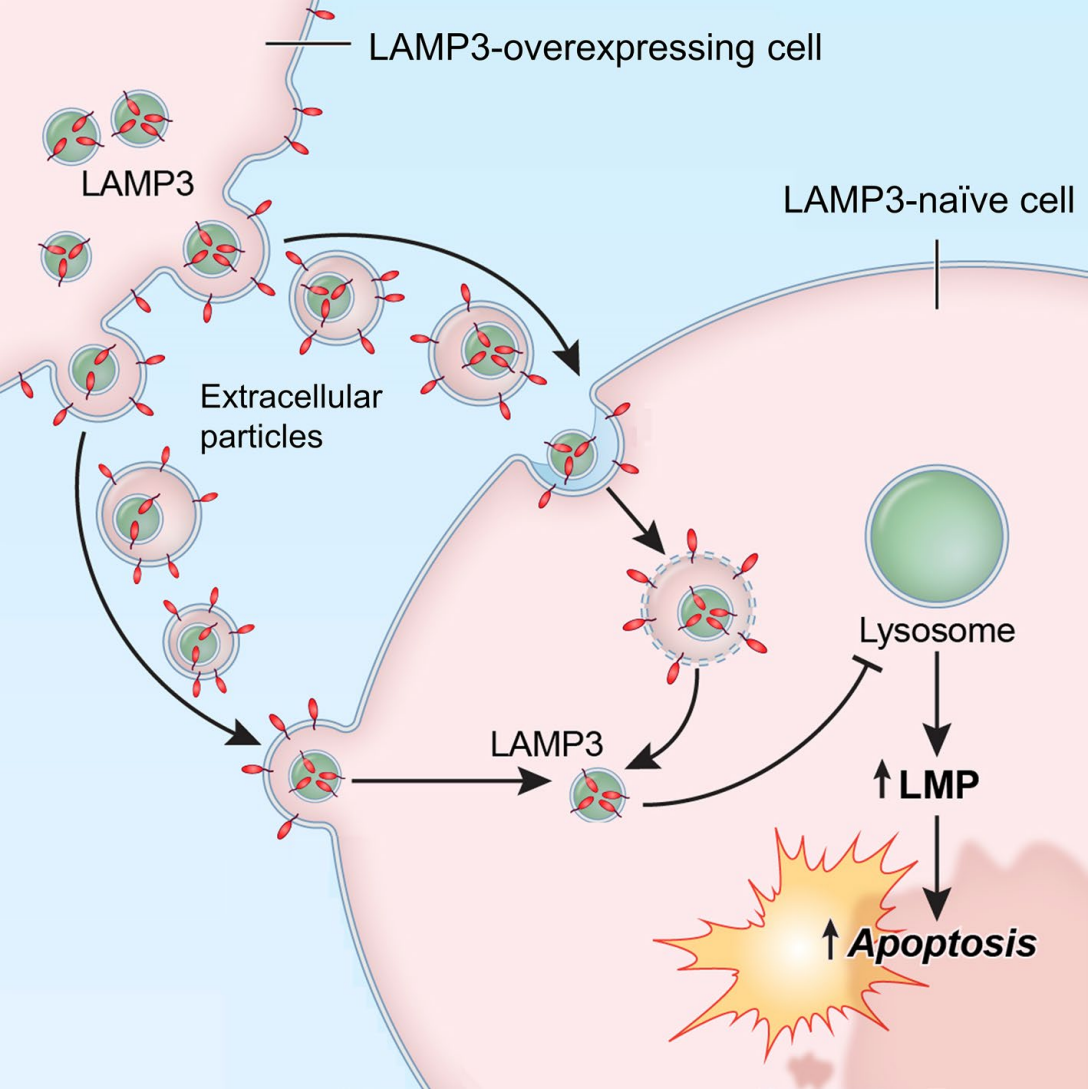
Graphical summary. Extracellular particles containing LAMP3 are released by LAMP3-overexpressing cells and taken up by a neighboring LAMP3-naïve cell. This results in LAMP3 protein transfer to the cytoplasm of the naïve cell. Here, LAMP3 induces lysosomal membrane permeabilization (LMP), leading to apoptotic cell death.

Taken together, LAMP3-overexpressing cells can transfer LAMP3 to a neighboring LAMP3-naïve cell via EPs, resulting in LMP and caspase-dependent apoptotic cell death in the naïve cell (Fig. 8).

## Discussion

EPs are increasingly being identified as a key pathway for transmitting warning signals to distant cells. This process of “dumping” particles and intracellular material can also contribute to cell survival by releasing the dysfunctional protein or organelles to prevent overwhelming the proteasomal and autophagic clearance pathways^32–34^. Cell death via apoptosis is often reported to be elevated in salivary gland epithelial cells of SjD patients, suggesting a loss of balance in the pathways associated with apoptosis and autophagy^4,35^.

We previously reported that LAMP3 expression is elevated in MSGs of SjD patients and that increased intracellular LAMP3 expression is associated with autoantigen release and cell death by caspase-dependent apoptosis^4,13^. What was not clear is how localized intracellular expression of LAMP3 can affect neighboring LAMP3-naïve cells of the LAMP3-expressing cells and more broadly affect overall gland activity. Confocal imaging of MSGs from SjD patients showed that LAMP3-positive material was being released from LAMP3-expressing cells. We tested the hypothesis that this material could be taken up by other cells and induce cellular changes within the recipient cells.

Further characterization showed that LAMP3 protein was abundantly present in the EPs from both stably and transiently LAMP3-overexpressing cells. EPs share surface markers with their parent cell^24^. LAMP3-overexpressing cells, as well as dendritic cells, express LAMP3 on their cell surface^36,37^. In accordance, we detected LAMP3 on the membrane surface of EPs derived from LAMP3-overexpressing A253 cells and on the cell surface of the EP producer cells.

Intercellular signal transfer via EPs can be initiated through interaction of the receptor on the plasma membrane of recipient cells with the ligand on the EP surface (such as programmed death-ligand 1 and its receptor^38^) and/or through transfer of the contents in EPs into the cytoplasm of recipient cells (such as caspase-1^39^) by the membrane fusion or endocytosis^40,41^. Cell-to-cell communication using this mechanism induces changes in the cellular function of the recipient cells. In our study, transfection of naïve A253 cells with rLAMP3 resulted in a significant increase in apoptotic cell death but treatment with rLAMP3 alone (without transfection reagent) did not. This suggests that LAMP3 present on the membrane surface cannot directly induce apoptosis but requires internalization.

Cleaved caspase-1 and cleaved caspase-3 trigger cell death. LAMP3 expression is known to induce the cleavage and activation of caspases^4,13^. Mitra et al*.* reported that mononuclear phagocytes undergoing apoptosis release cleaved caspase-1 via EPs and induce apoptosis in pulmonary vascular endothelial cells receiving the cleaved caspase-1-containing EPs^39^. As shown in the present paper, apoptosis in naïve cells induced by LAMP3-associated EPs was sensitive to treatment with the pan-caspase inhibitor zVAD-fmk, suggesting this apoptosis was caspase dependent. However, proteomics showed no significant increases in cleaved caspase-1 and caspase-3 in EPs from LAMP3-overexpressing cells. This indicates that apoptosis induced by LAMP3-associated EPs was independent of EP-derived exogenous caspase proteins but does depend on endogenous caspases activated by transferred LAMP3 protein.

LAMP3 is a glycosylated protein^42^. The variation in size of LAMP3 in the present study is more likely the result of differences in glycosylation. We did not see any association between the size of LAMP3 and apoptosis. However, we could observe apoptosis in cells transfected with recombinant LAMP3, which had limited glycosylation, suggesting that glycosylation was not required for LAMP3-induced apoptosis.

We demonstrated for the first time cell-to-cell transfer of LAMP3 via EPs in salivary gland epithelial cell line. The data presented in this study support the notion that changes in epithelial cells can be transmitted to other epithelia and possibly to immune cells in the gland via EPs. In support of our findings, EPs from irradiated-tumor cells have been shown to contain reactive oxygen species and can induce cell death via ferroptosis in these tumor cells^43^. In addition, Barbati et al*.* reported that EPs from peripheral blood mononuclear cells of patients with rheumatoid arthritis induce apoptosis in endothelial cells^44^. As LAMP3 contributes to antigen presentation by antigen-presenting cells^45^, it is possible that LAMP3 in EPs contributes to activation of the immune response within the salivary glands. Clarification of the cross talk between LAMP3-associated EPs and immune cells is required to better understand this aspect of the pathogenesis in SjD. Additional in vitro and in vivo work using model organism will be necessary to further validate these findings.

In summary, we showed that LAMP3 induced the increase in apoptosis through EP-mediated cell-to-cell communication. These results define a broader role of LAMP3 in the salivary glands of SjD patients. The findings provide support for targeting inhibition of LAMP3 expression and function as a therapeutic approach in the treatment of SjD.

## Methods

### Human subjects

Patients with SjD diagnosed based on the 2002 American-European Consensus Group criteria^46^ were recruited at the National Institutes of Health in accordance with the Declaration of Helsinki principles. All the protocols were reviewed and approved by the National Institutes of Health Institutional Review Board (NCT00001390, NCT02327884, and NCT00001196). All participants provided informed consent prior to the initiation of any study procedure.

### Cells and plasmids

A253 cells (ATCC, Manassas, VA, USA), a cell line of salivary ductal cell origin, were cultured in McCoy’s 5A medium (Thermo Fisher Scientific, Waltham, MA, USA) supplemented with 10% FBS at 37 °C in 5% CO_2_.

Plasmids pCMV6-LAMP1 (SC116652), pLenti-LAMP3-Myc-DDK (RC205344L3), and pLenti-LAMP3-mGFP (RC205344L4) were purchased from OriGene (Rockville, MD, USA). Plasmid pME18S-LAMP3 and its control empty plasmid (pME18S) were built as described previously^4,47^. All plasmids were purified using the EndoFree Plasmid Maxi Kit (QIAGEN, Valencia, CA, USA). To establish A253-LAMP3 and A253-LAMP3-mGFP cells, A253 cells were transfected with pLenti-LAMP3-Myc-DDK or pLenti-LAMP3-mGFP, respectively, using Lipofectamine 3000 (Life Technologies, Waltham, MA, USA) and stably propagated under puromycin selection (1 μg/mL).

### Treatment of LAMP3-naïve cells with cell culture medium

Cells (5 × 10^5^ cells) were transfected with a total amount of 1.5 μg control, *LAMP3*-encoding or *LAMP1*-encoding plasmid using Lipofectamine 3000. After 24 h, 5 × 10^5^ transfected cells were replated in one well of a 6-well plate with 2 mL of medium supplemented with 10% exosome-depleted FBS (Thermo Fisher Scientific). After 48 h, culture medium was collected and centrifuged at 2000*g* at 4 °C for 30 min to eliminate cells and debris. Culture medium was separated to pellet and supernatant by additional ultracentrifugation at 100,000*g* at 4 °C for 2 h. Thereafter, 0.5 × 10^5^ naïve cells were cultured with 2 mL of the collected medium, the pellet resuspended in 2 mL of medium, or 2 mL of supernatant. Cells were used for apoptosis assay after 48 h.

### Separation of extracellular particles

Cells (2.5 × 10^6^ cells) were replated on a 10-cm dish with 10 mL of medium supplemented with 10% exosome-depleted FBS. After 72 h, culture medium was collected and centrifuged at 2000*g* at 4 °C for 30 min to eliminate cells and debris. The supernatant was additionally centrifuged at 10,000*g* at 4 °C for 60 min^48^. The pellet was resuspended in PBS and named EP fraction 1 (EP-f1). The supernatant was incubated with Total Exosome Isolation Reagent (Thermo Fisher Scientific) at 4 °C overnight. The mixture was centrifuged at 10,000*g* at 4 °C for 60 min. The pellet was resuspended in PBS and named EP fraction 2 (EP-f2).

### Treatment of LAMP3-naïve cells with extracellular particles

Total protein concentration in EPs was quantified using Bradford assay (Thermo Fisher Scientific). LAMP3-naïve cells were washed twice in medium supplemented with 10% exosome-depleted FBS. Next, 5 × 10^4^ cells were incubated with 2 μg of EPs in a well of a 6-well plate with 2 mL of medium supplemented with 10% exosome-depleted FBS. Heat treatment of EPs was performed at 100 °C for 30 min. Cells were used for Western blot analysis after 6 h and for apoptosis assay after 72 h.

### Proteomic analysis

EPs (fraction 1 and fraction 2) derived from LAMP1-overexpressing and LAMP3-overexpressing A253 cells were treated with 8 M urea to disrupt membranes and dissolve proteins. Proteins were reduced with 2.5 mM dithiothreitol (DTT) and cysteine alkylated with 25 mM methyl methanethiosulfonate. The resulting solution was diluted with 0.1 M triethylammonium bicarbonate (TEAB) to reduce the urea concentration to 4 M. To each sample, 5 µg Trypsin/Lys-C Mix (Promega, Madison, WI, USA) was added, and samples were incubated at 37 °C for 3 h. The digestion solution was further diluted with 0.1 M TEAB to 0.8 M urea and incubated at 37 °C overnight. Tryptic digests were acidified with trifluoroacetic acid (TFA) to pH 4 and desalted using the C18 Spin Column (TT2 C18) (Glygen Corp., Columbia, MD, USA). Eluents from the C18 spin columns were vacuum dried and dissolved in 50 µL 0.05% TFA. Peptide amount was normalized based on reading at UV 280 nm before analysis.

Nano-LC–MS/MS analysis was carried out with the Obitrap Fusion Lumos Tribrid Mass Spectrometer interfaced with the UltiMate 3000 RSLCnano HPLC System (both purchased from (Thermo Fisher Scientific) according to the protocol described previously in Ref.^49^. Briefly, tryptic digest (1 µg) was loaded and desalted in an ZORBAX 300 SB-C18 Trapping Column (0.3 × 5 mm; Agilent, Santa Clara, CA, USA) at 5 μL/min for 5 min. Peptides were then eluted from a 75 μm × 250 mm Acclaim PepMap 100 column (3 μm, 100 Å; Thermo Fisher Scientific) and chromatographically separated using a binary solvent system consisting of solvent A (0.1% formic acid and 2.5% acetonitrile) and solvent B (0.1% formic acid and 80% acetonitrile) at a flow rate of 300 nL/min. A gradient was run from 1% solvent B to 42% solvent B for 150 min, followed by a 5-min wash step with 80% solvent B and a 10-min equilibration at 1% solvent B before the next sample was injected. Precursor masses were detected using the Orbitrap Fusion Lumos Tribrid Mass Spectrometer at R = 120,000 (*m/z* 200). Fragment masses were detected in the linear ion trap at unit mass resolution. Data-dependent MS/MS was performed with top of speed setting; cycle time was 3 s with dynamic exclusion of 30 s.

Protein identification and label-free quantification were carried out using the Proteome Discoverer software package (Thermo Fisher Scientific). Raw data were searched against a human proteome database from UniProt (2019-05-15) along with a contaminant protein database with both Sequest HT and Mascot search engines. Cysteine alkylation with methylthio (+ 45.988) was set as a fixed modification. Methionine oxidation, asparagine, and glutamine deamidation were set as variable modifications. Peptide mass tolerance was set to ± 20 ppm for the database search and was later filtered to 5 ppm for report; fragment mass tolerance was ± 0.6 Da. For relative quantification, label-free quantification was performed using the MINORA Feature Detection node followed by precursor intensity quantification of unique and razor peptides. Normalization between samples was carried out based on total peptide amount. Changes above two-fold were considered significant (based on ANOVA).

### Transfection of recombinant proteins

Recombinant LAMP3 (recombinant DC-LAMP, rLAMP3), recombinant LAMP1 (rLAMP1) and recombinant EGFP (rEGFP) were purchased from Novus Biological (Centennial, CO, USA). Control FLAG peptide was purchased from Thermo Fisher Scientific. Heat treatment of rLAMP3 was performed at 100 °C for 30 min. Each recombinant protein was introduced into cells using Xfect Transfection Reagent (Takara Bio, Mountain View, CA, USA). Cells were used for Western blotting after 14 h and for apoptosis assay after 14 h.

### Immunofluorescence for LAMP3 expression in patients’ minor salivary glands

Tissue sections (5 μm) were cut from formalin-fixed, paraffin-embedded blocks of MSG biopsies as described previously^4^. These sections were placed on glass slides, deparaffinized, rehydrated, and subjected to citric acid microwave antigen retrieval. Slides were first blocked with 5% donkey serum (Jackson ImmunoResearch Laboratories, West Grove, PA, USA) and 0.5% BSA (Sigma-Aldrich Corp., The Woodlands, TX, USA) in PBS at 25 °C for 1 h in a humidified chamber and then incubated with 100 μL of 10 μg/mL anti-LAMP3 polyclonal antibody (12632-1-AP; Proteintech, Rosemont, IL, USA) at 4 °C overnight. The next day, slides were washed in five changes of PBS for 5 min each and then incubated with 1:200 dilution of 2 mg/mL Alexa Fluor 488 AffiniPure Donkey Anti-Rabbit IgG (Cat. 711-545-152; Jackson ImmunoResearch Laboratories) at 25 °C in the dark for 1 h, followed by washing in five changes of PBS for 5 min each and counterstaining with DAPI mounting medium.

All images were acquired by using the FV1000 confocal microscope and software (Olympus, Center Valley, PA, USA). Analysis and quantification of expression were performed by using Icy v.1.9.10.0 (BioImage Analysis Unit, Institut Pasteur, Paris, France).

### Immunofluorescence for galectin-3 puncta formation

Galectin-3 staining was performed according to the protocol described previously^13^. Briefly, cells were fixed by treatment with 4% paraformaldehyde/PBS at 22 °C for 15 min and washed twice in PBS for 5 min each. Fixed cells were permeabilized by treatment with 0.1% Triton-X 100 in PBS at 22 °C for 10 min and then incubated with 2% BSA/PBS at 22 °C for 30 min. After blocking, cells were incubated with 10 μg/mL Mouse anti-Galectin-3 Monoclonal Antibody (Abcam) in 2% BSA/PBS at 4 °C overnight. Cells were washed three times in PBS for 10 min each and then incubated with 10 μg/mL Alexa Fluor 647 anti-Mouse IgG (Jackson ImmunoResearch Laboratories) in 2% BSA/PBS at 22 °C for 1 h. Thereafter, cells were washed three times in PBS for 10 min each and then mounted with DAPI mounting medium (Abcam). Stained cells were analyzed by using a Nikon fluorescent microscope and quantified by using ImageJ software.

### Live-cell imaging

For EP release experiments, A253-LAMP3-mGFP cells were plated on 35-mm glass bottom culture dishes (MatTek Corporation, Ashland, MA, USA) with phenol-red free McCoy’s 5A medium (Cytiva, Marlborough, MA, USA) supplemented with 10% FBS, 24 h before imaging experiments. One hour before cells were imaged, culture medium was replaced with fresh phenol-red free McCoy’s 5A medium (Cytiva). Individual cells were imaged using either a Nikon A1 HD25 confocal microscope with a CFI Plan Apo IR 60×/1.27 NA water immersion objective or a Nikon CSU-X1 Spinning Disk microscope equipped with a Lambda S 100XC silicone immersion objective (Nikon) and Photometrics Prime 95B camera (TELEDYNE PHOTOMETRICS. Tucson, AZ, USA). Humidified live imaging environments were maintained at 37 °C and 5% CO_2_ for the duration of each experiment. Time-lapse acquisition of cell volumes was performed using NIS-Elements software (Nikon).

For EP uptake experiments, naïve A253 cells were plated on 35-mm glass bottom dishes (MatTek Corporation) with phenol-red free McCoy’s 5A medium (Cytiva) 24 h before imaging experiments. One hour before cells were imaged, culture medium was replaced with fresh phenol-red free McCoy’s 5A medium (Cytiva). Immediately before the start of image acquisition, 100 μL of LAMP3-mGFP–containing EPs were added to the naïve cells. Individual fields of view were imaged using a Nikon TiE widefield microscope with a Plan Fluor 40×/0.75 NA objective (Nikon) and Hamamatsu Orca-Flash 4.0 camera (HAMAMATSU PHOTONICS K.K., Bridgewater, NJ, USA). Humidified live imaging environments were maintained at 37 °C and 5% CO_2_ for the duration of each experiment. Image acquisition was controlled by NIS-Elements software.

### Western blot

Western blotting was performed according to a previously described protocol^4^. The following primary antibodies were used: anti-LAMP3 and anti-LAMP1 polyclonal antibodies (both purchased from Proteintech), and anti-FLAG, anti-α-tubulin, and anti-β-actin monoclonal antibodies (all three purchased from Sigma-Aldrich, St. Louis, MO, USA). The following secondary antibodies were used: Mouse IgG HRP-Linked Whole Antibody (GENA931) and Rabbit IgG HRP-Linked Whole Antibody (GENA934) (both purchased from Sigma-Aldrich). To confirm whether proteins were present, membranes were stained by using Reversible Protein Stain Kit (Thermo Fisher Scientific).

### Apoptosis assay

zVAD-fmk was purchased from Enzo Life Sciences. Apoptosis analysis of transfected cells was described previously^4^. Briefly, treated A253 cells in all experiments were collected using trypsin digestion and washed twice in ice-cold PBS. Apoptotic cells were stained using the APC Annexin V Apoptosis Detection Kit with 7-AAD (BioLegend, San Diego, CA, USA) according to the manufacturer’s instructions. Stained cells were detected using the BD Accuri Flow Cytometer (BD Biosciences, San Jose, CA, USA) and analyzed using FlowJo software (BD Biosciences).

### Immunoprecipitation to detect LAMP3 expression on membrane surface of extracellular particles

EPs collected from LAMP3-overexpressing and control A253 cells were incubated at 4 °C overnight with exosome isolation magnetic beads (Thermo Fisher Scientific), which were conjugated with biotin anti-LAMP3 monoclonal antibody (clone:104G4; Novus Biological) according to the manufacturer’s instructions. After washing in 0.1% BSA in PBS, beads were incubated with anti-LAMP3 polyclonal antibody or normal rabbit IgG (Sigma-Aldrich) at 25 °C while gently shaking for 1 h. Thereafter, beads were washed in 0.1% BSA in PBS and incubated with 10 μg/mL Alexa Fluor 647 anti-Rabbit IgG (A-21244, Thermo Fisher Scientific) at 25 °C while gently shaking for 40 min. After washing, stained beads were detected using the BD Accuri Flow Cytometer (BD Biosciences) and analyzed using FlowJo software (BD Biosciences).

### Statistical analysis

Student’s *t*-test was used to compare two groups and one-way ANOVA to compare more than two groups. These analyses were carried out with GraphPad Prism 8 (GraphPad Software, San Diego, CA, USA). A *P-*value < 0.05 was regarded as statistically significant. All statistical tests were two-sided.

## Supplementary Information

Supplementary Video 1.

Supplementary Video 2.

Supplementary Information.

## Data Availability

The datasets generated and/or analyzed during the current study are available in the PRIDE repository (accession number: PXD036774).
