# Supplementary material for: LAMP3 transfer via extracellular particles induces apoptosis in Sjögren’s disease
Source: Sci Rep. 2023 Feb 14;13:2595. doi: 10.1038/s41598-023-28857-w (PMC9929273; doi:10.1038/s41598-023-28857-w)

LAMP3 transfer via extracellular particles induces apoptosis in Sjögren's disease

Tsutomu Tanaka[1†], Hiroyuki Nakamura[1†], Duy T. Tran[2], Blake M. Warner[3], Yan Wang[4], Tatsuya Atsumi[5], Masayuki Noguchi[6], John A. Chiorini[1*]

1. Adeno-Associated Virus Biology Section, National Institute of Dental and Craniofacial Research, National Institutes of Health, Bethesda, MD, USA.
2. NIDCR Imaging Core, National Institute of Dental and Craniofacial Research, National Institutes of Health, MD, USA.
3. Salivary Disorders Unit, National Institute of Dental and Craniofacial Research, National Institutes of Health, Bethesda, MD, USA.
4. Mass Spectrometry Facility, National Institute of Dental and Craniofacial Research, National Institutes of Health, Bethesda, MD, USA.
5. Department of Rheumatology, Endocrinology and Nephrology, Faculty of Medicine and Graduate School of Medicine Hokkaido University, Sapporo, Japan.
6. Division of Cancer Biology, Institute for Genetic Medicine Hokkaido University, Sapporo, Japan.

† These authors contributed equally.

* Author to whom correspondence should be addressed:

Dr. John A. Chiorini, PhD

Adeno-Associated Virus Biology Section/National Institute of Dental and Craniofacial Research, National Institutes of Health

10 Center Drive, Bethesda, MD 20892, USA

Phone: 301-496-4279, Fax: 301-402-1228, Email: jchiorini@dir.nidcr.nih.gov

Supplementary Table 1. Abundance of marker proteins in extracellular particles.

Protein name	LAMP1 EP-f1	LAMP3 EP-f1	LAMP1 EP-f2	LAMP3 EP-f2
CD9	35645633	34843101	11730.95	-
CD63	1003532	1407335	-	-
CD81	3771648	5401073	3438062	3504295
TSG101	380669.5	290319.9	-	-
ALIX (PDCD6IP)	20297187	21675386	8614077	7871078
Calnexin	30578777	53290352	65190.08	28152.63

Supplementary Table 2. Enriched proteins (> 2-fold increase) in extracellular particle fraction 1 derived from LAMP3-overexpressing cells.

Protein name	Relative change in expression		
	vs LAMP1 EP-f1	vs LAMP1 EP-f2	vs LAMP3 EP-f2
**LAMP3**	**48.3**	**118.3**	**9.2**
ATP6V1C1	6.1	21.0	39.0
STX6	4.2	2.3	2.1
PCBP2 (fragment)	3.7	3.6	4.3
Protein S100-A2	3.2	20.8	31.5
MTOR	2.8	6.4	8.4
ALG5	2.5	25.4	56.6
PGRMC1	2.5	2758.9	1914.3
LTN1	2.3	3.1	2.8
EIF2B5	2.1	3.3	3.2
Basal-cell adhesion molecule	2.1	24.3	58.7
hnRNP H2	2.1	17.0	66.1

EP-f1, extracellular particle fraction 1; EP-f2, extracellular particle fraction 2.

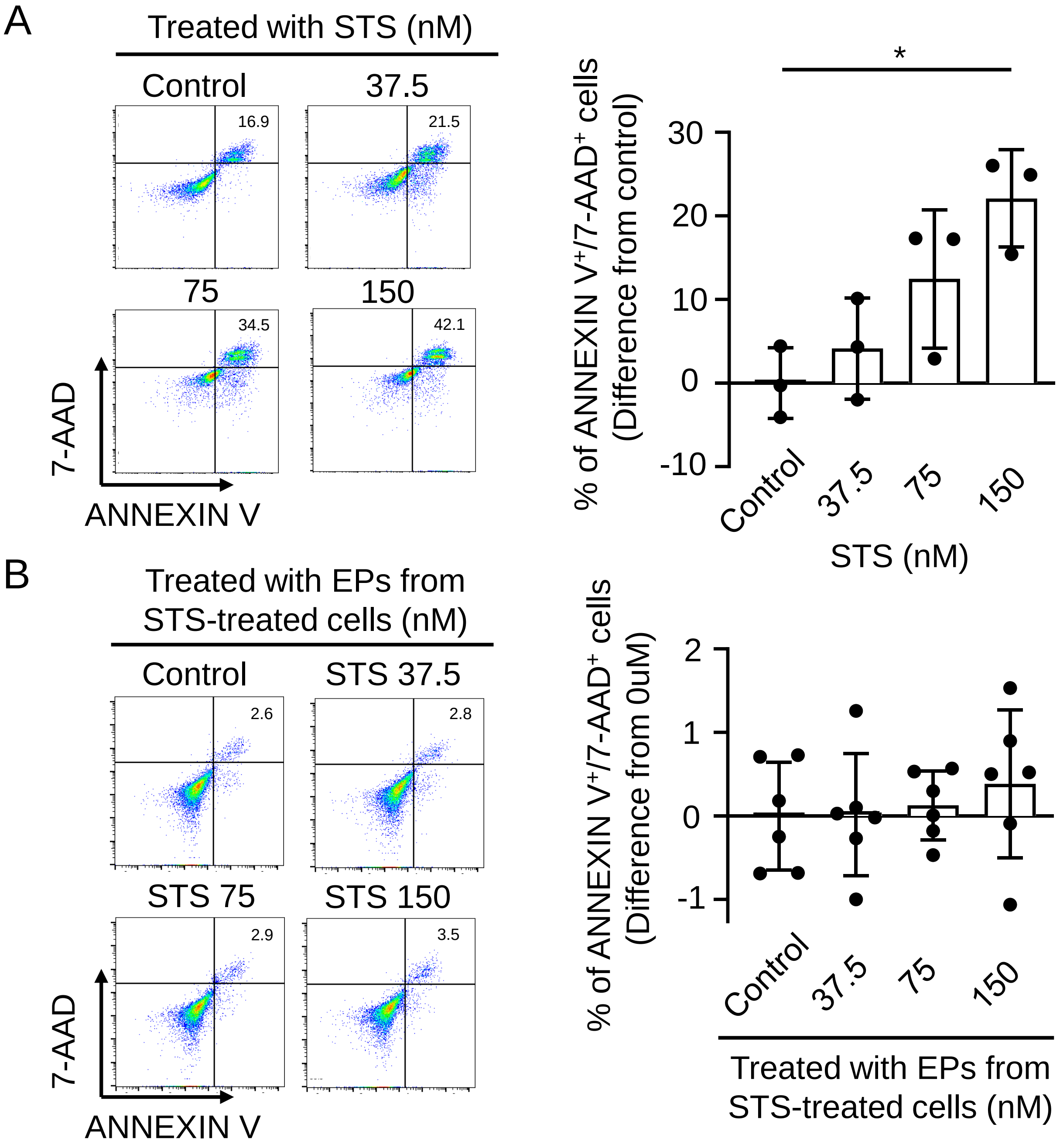

Supplementary Fig. 1. Extracellular particles released from staurosporine-treated A253 cells do not induce apoptosis (A) Naïve A253 cells were treated with apoptosis inducer staurosporine (STS) for 72 hours (*n* = 3). **(B)** Naïve A253 cells were treated with extracellular particles (EPs) released from STS-treated A253 cells for 72 hours (*n* = 6). Number of apoptotic cells was determined by flow cytometry using APC Annexin V/7-AAD. Graphs showing difference in mean (± SD) number of Annexin V^{+}/7-AAD^{+} cells in naïve A253 cell culture compared with control.

*$p < 0.05$ (one-way ANOVA)

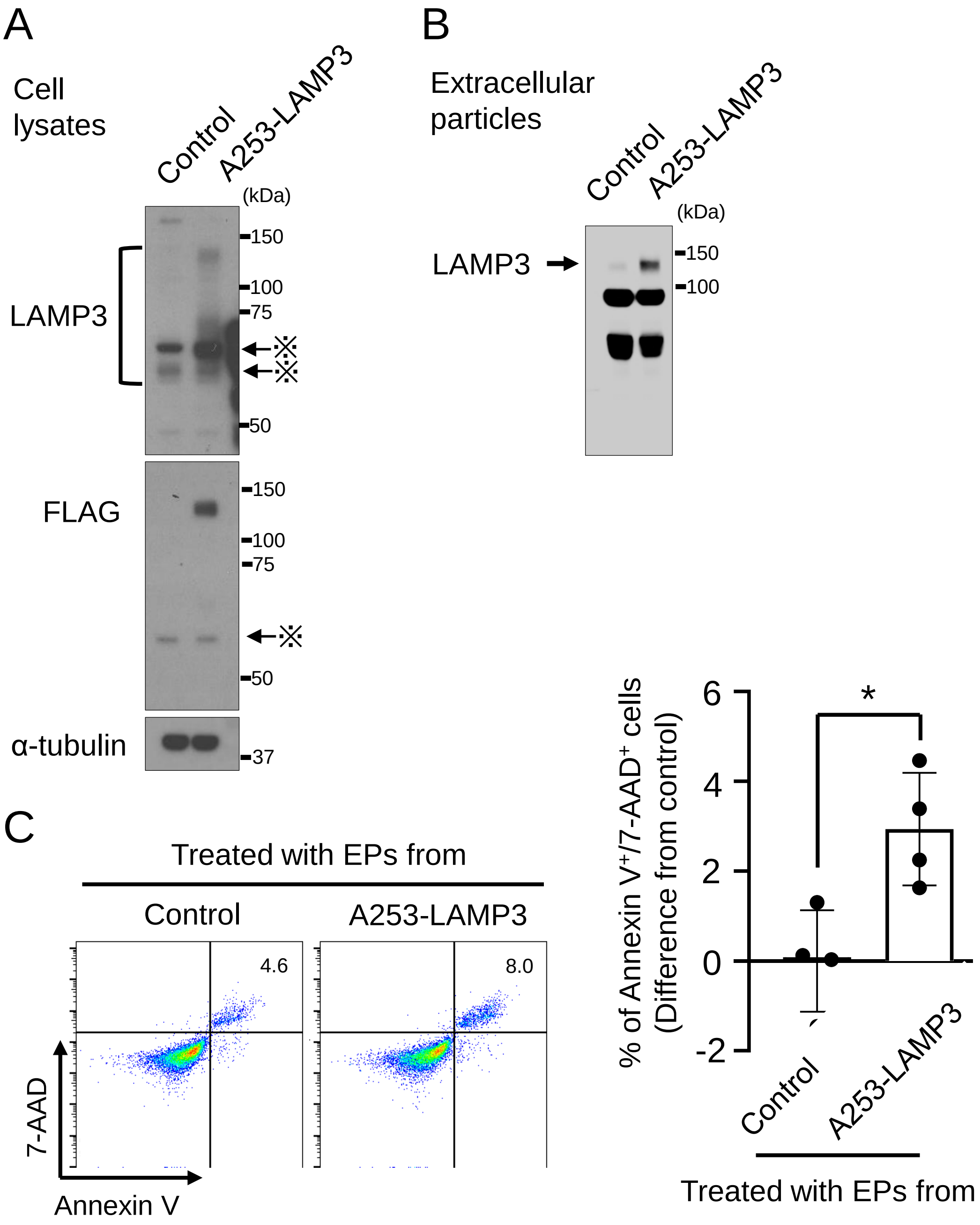

Supplementary Fig. 2. Extracellular particles derived from A253 cells stably overexpressing LAMP3 induce apoptosis in naïve A253 cells. (A) Western blot analysis of control A253 cells and A253 cells stably overexpressing LAMP3-FLAG fusion protein (A253-LAMP3 cells). "※" indicates non-specific bands. **(B)** Western blot analysis of equal volume of extracellular particles (EPs) released from control and A253-LAMP3 cells. **(C)** Naïve A253 cells were treated with EPs from control or A253-LAMP3 cells. Number of apoptotic cells in naïve-cell culture was determined by flow cytometry using APC Annexin V/7-AAD 72 hours after incubation. Graph showing difference in mean (± SD) number of Annexin $V^+/7\text{-}AAD^+$ cells in naïve A253 cell culture treated with EPs derived from A253-LAMP3 cells compared with EPs derived from control A253 cells ($n = 4$).

*$p < 0.05$ (unpaired Student's t-test)

Supplementary Fig 3. Original uncropped gels.

Figure 2A

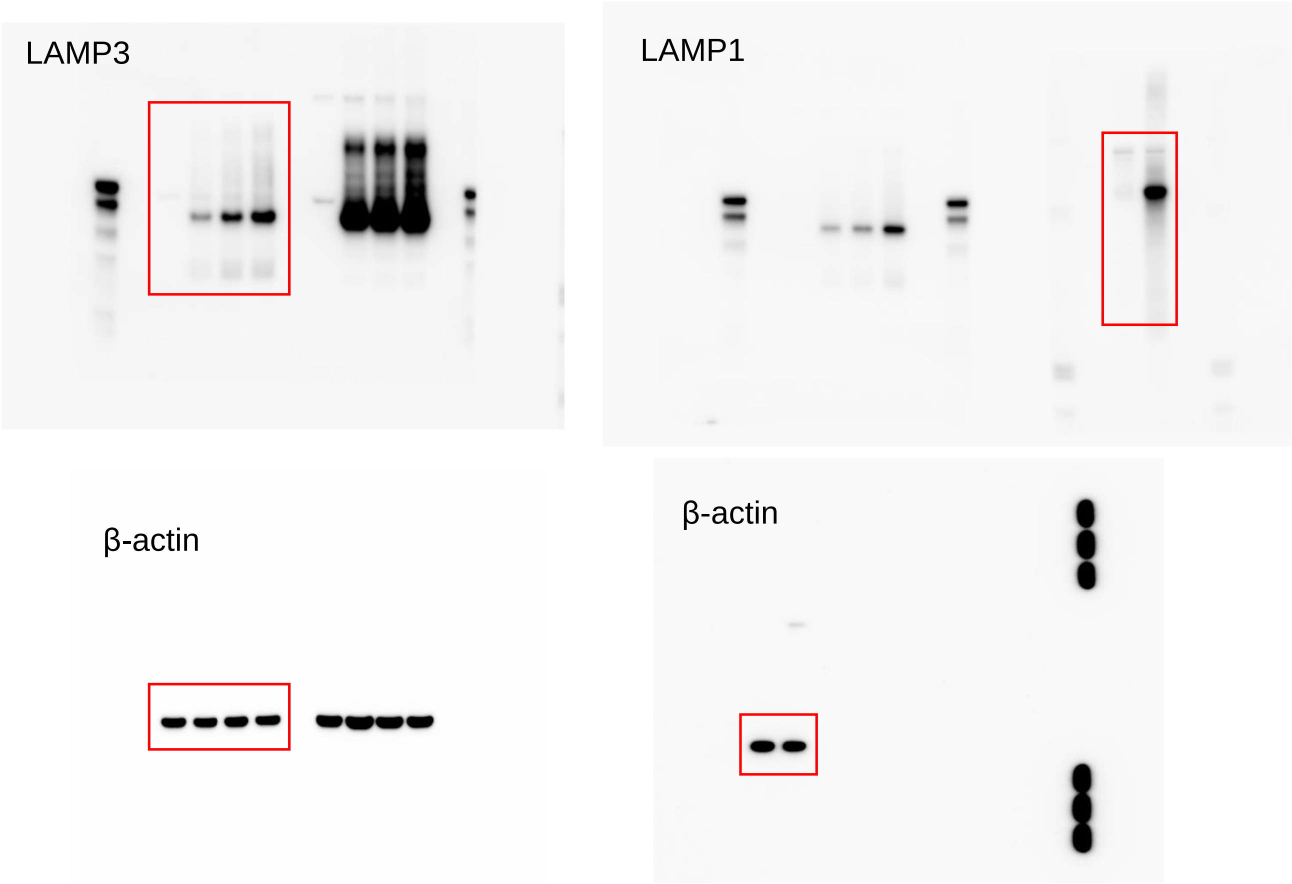

Supplementary Fig 3. Original uncropped gels.

Figure 4A

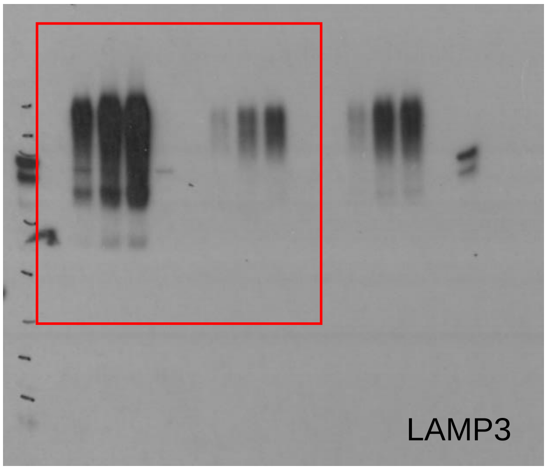

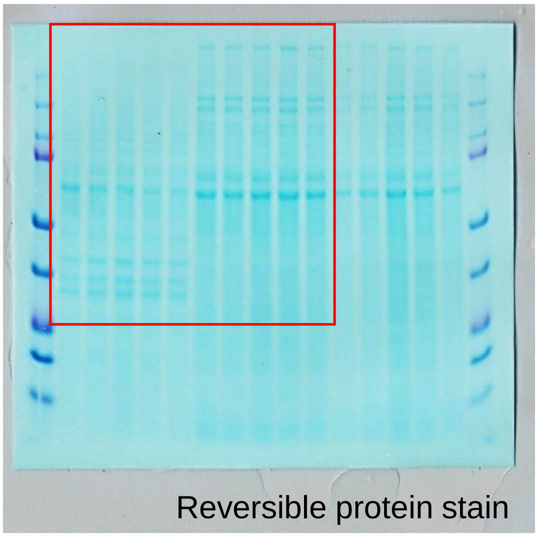

Figure 5A

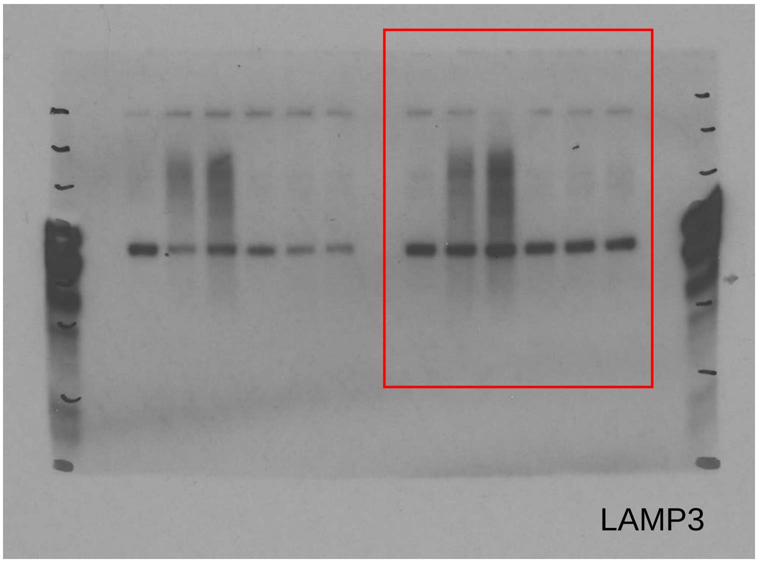

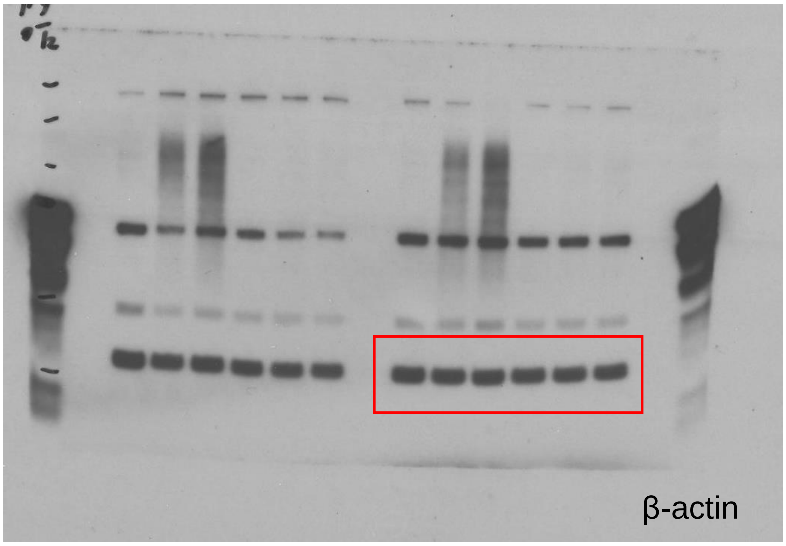

Supplementary Fig 3. Original uncropped gels.

Figure 5B

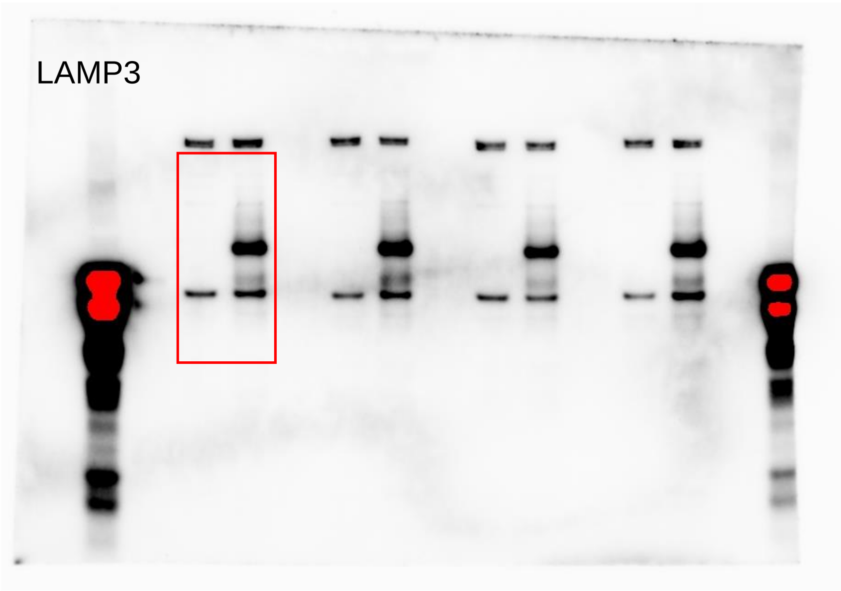

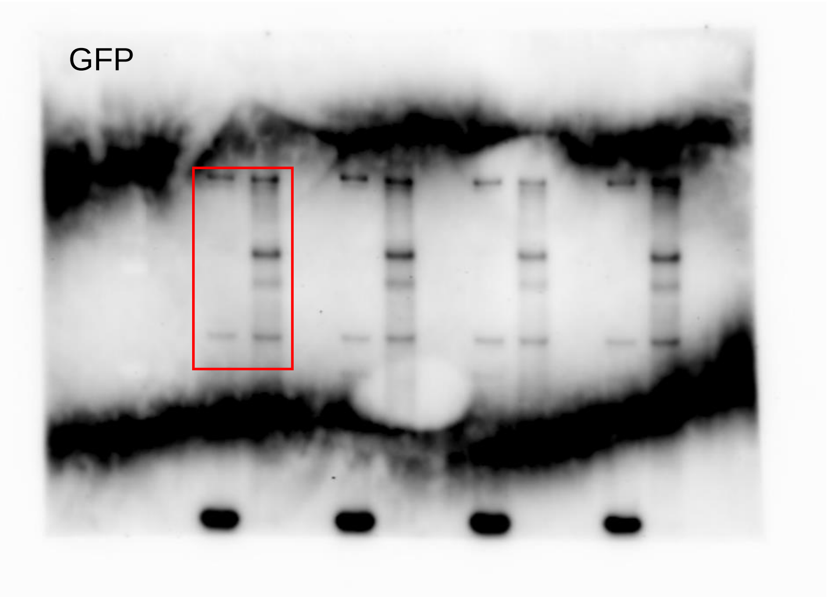

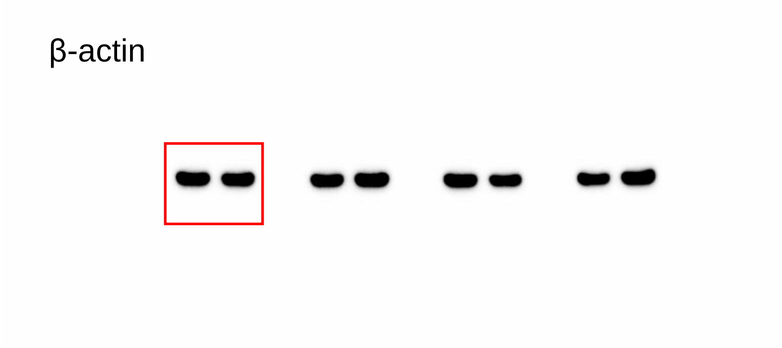

Figure 5C

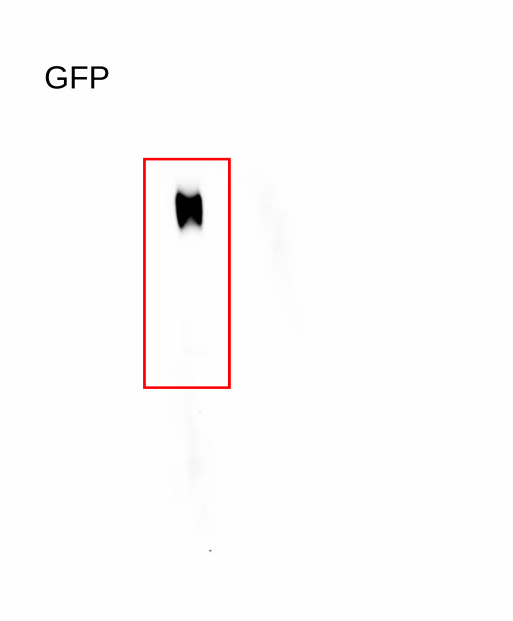

Supplementary Fig 3. Original uncropped gels.

Figure 6A

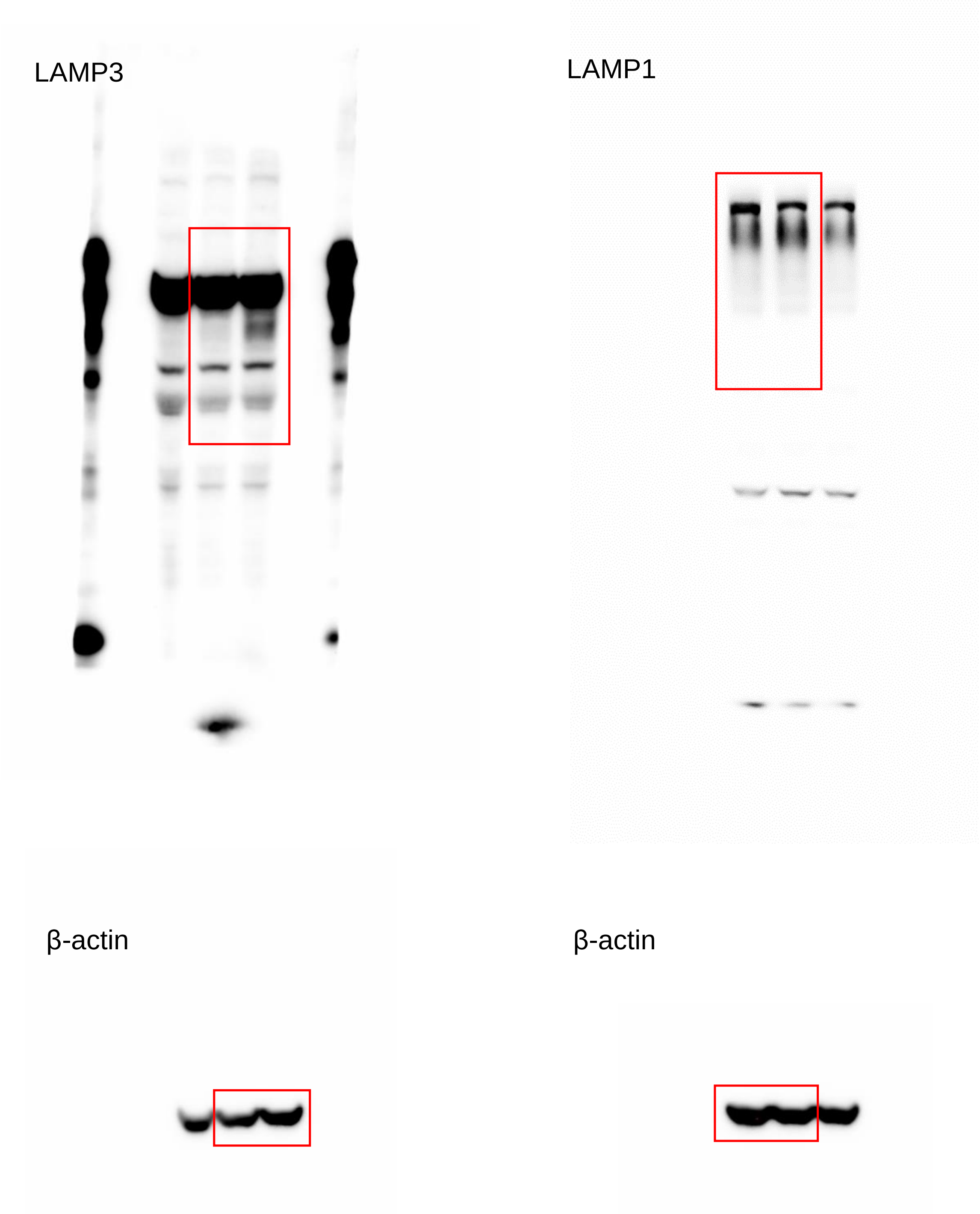

Supplementary Fig 3. Original uncropped gels.

S-Figure 2A

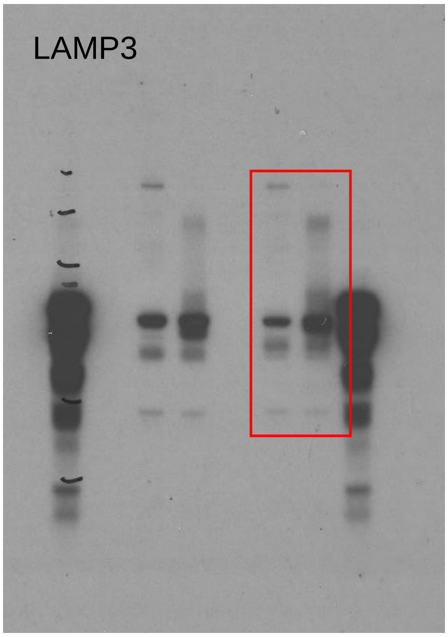

S-Figure 2B

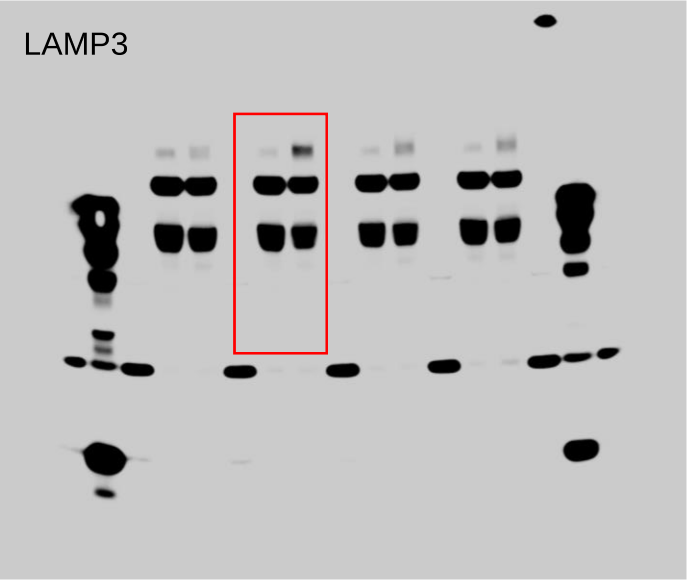

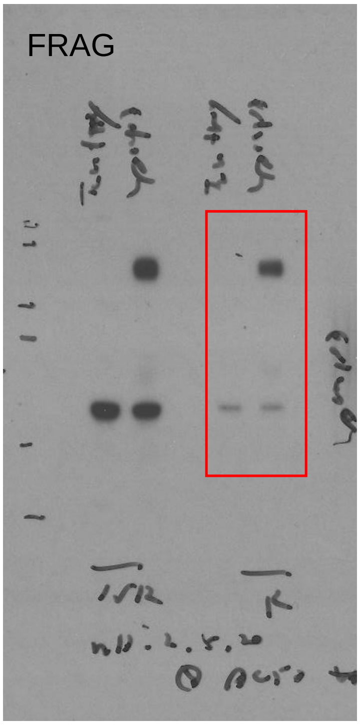

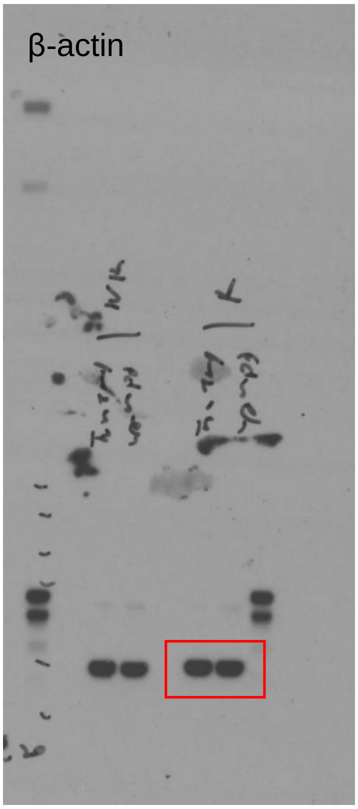

Supplement: Supplementary file 3 — Supplementary Information. [file 41598_2023_28857_MOESM3_ESM.pdf]
